# ABS-FishCount: An Agent-Based Simulator of Underwater Sensors for Measuring the Amount of Fish

**DOI:** 10.3390/s17112606

**Published:** 2017-11-13

**Authors:** Iván García-Magariño, Raquel Lacuesta, Jaime Lloret

**Affiliations:** 1 Department of Computer Science and Engineering of Systems, University of Zaragoza, 44003 Teruel, Spain; lacuesta@unizar.es; 2 Instituto de Investigación Sanitaria Aragón, University of Zaragoza, 50009 Zaragoza, Spain; 3 Integrated Management Coastal Research Institute, Universitat Politècnica de València, 46022 València, Spain; jlloret@dcom.upv.es

**Keywords:** agent-based simulation, agent-based social simulation, multi-agent system, agent-oriented software engineering, underwater sensor, underwater sensor network, simulator software, fish measurement

## Abstract

Underwater sensors provide one of the possibilities to explore oceans, seas, rivers, fish farms and dams, which all together cover most of our planet’s area. Simulators can be helpful to test and discover some possible strategies before implementing these in real underwater sensors. This speeds up the development of research theories so that these can be implemented later. In this context, the current work presents an agent-based simulator for defining and testing strategies for measuring the amount of fish by means of underwater sensors. The current approach is illustrated with the definition and assessment of two strategies for measuring fish. One of these two corresponds to a simple control mechanism, while the other is an experimental strategy and includes an implicit coordination mechanism. The experimental strategy showed a statistically significant improvement over the control one in the reduction of errors with a large Cohen’s d effect size of 2.55.

## 1. Introduction

The water covers more than 70% of the area of our planet. Underwater sensors have become a useful technology for exploring the underwater life and other aspects. The advances of physical underwater sensors have allowed monitoring a high number of environmental parameters [1]. In this context, these sensors usually communicate among each other for conforming underwater sensor networks [2], the acoustic networks being the most common ones. Acoustic underwater sensor networks are useful for real-time communications even in networks with a low density of nodes [3]. Simulation tools can be useful for testing and analyzing certain approaches of underwater sensor networks in the early stages without needing the high costs of deploying an actual underwater sensor network [4].

There are many different kinds of underwater sensors such as cameras [5], depth sounder transducers [6] and chemical-based sensors [7]. In the case of cameras, some works propose algorithms for underwater object detection with different techniques from the field of computer vision (CV) [8]. Even some other sensors have been used from outside water to observe the fish such as the Microsoft Kinect devices (Nové Hrady, Czech Republic) in controlled experiments [9].

The measurement of fish and their qualities can be useful for (a) understanding ecosystems in order to avoid the extinction of some species by monitoring the fish census [10], (b) knowing the production of fish farms in advance to plan the right amount of sales [11], (c) reducing the amount of uneaten food in marine fish farms for increasing their benefits [12], and (d) analyzing which are the better conditions for assuring the health of fish [13]. The most common sensors for counting fish are the Dual-frequency IDentification SONar (DIDSON) sonars [14]. DIDSON sonars provide images of the underwater objects, and these can be analyzed using CV techniques [15] in order to detect fish.

Marine fish farms can benefit from the use of underwater sensors and underwater sensor networks. The information of sensor networks can be used to properly feed fish when appropriate in these fish farms [16]. There is simulator software that allows designing and simulating different scalable underwater wireless sensors for achieving the sustainability of fish farms [17]. Group-based sensor networks can be useful for monitoring the sustainability of marine fish farm, by controlling the feed and fecal waste in the seabed under the cages [18]. The performance of the group-based sensor networks relies on the appropriate way of placing and moving these underwater sensors.

In this research field, multi-agent systems (MASs) have been useful for managing underwater sensor networks and monitoring underwater scenarios. For instance, an MAS was used for routing underwater acoustic sensor networks [19]. Another MAS implemented different reconfigurable control strategies for switching topology networks [20]. A different MAS was developed for the early detection of aquatic pests [21]. Agent-based simulators (ABSs) are a specific kind of MAS that are specifically intended for simulating and reproducing certain initial circumstances to estimate the evolution and final results after an elapsed time [22].

In this context, the current work presents an ABS named ABS-FishCount and its underlying framework for the definition of fish measurement strategies. This ABS allows for assessing different fish measurement strategies by comparing the number of fish with the amount estimated by the strategies. This work simulates the use of depth acoustic underwater sensors for detecting fish with a low error rate. This simulator is called ABS-FishCount, and uses grids of sensors that can slightly vary their position. One of the main goals of these strategies is the coordination for obtaining an accurate global collective measurement.

The remainder of the article is organized as follows. The next section introduces the most relevant related works. Section 3 presents the novel ABS for defining and assessing strategies for measuring fish. Section 4 illustrates the current approach by experiencing and comparing two different measurement strategies, i.e., a simple one used as control mechanism and an experimental one with an implicit coordination mechanism. Section 5 mentions the conclusions, and introduces possible future lines of research.

## 2. Related Work

### 2.1. Measurement of Fish and Their Properties

Fish properties are mostly measured by underwater sensors. These sensors can monitor fish from a certain distance [13] or in some cases are attached to the fish [7]. The most commonly measured fish properties are stress and bioenergetics.

Regarding the measurement of fish stress, Simon et al. [13] presented an approach based on the analysis of fish movements. It improved the alternative methods, in the sense that it did not require taking the fish out of the water, which is quite stressful for them. Shinoda et al. [7] also measured the fish stress. In this case, they used chemical-based sensors in devices attached to the fish. These devices used light-based transmission of data for communicating of the monitoring results. This transmission was possible in seawater, in which radio waves would be difficult to communicate.

Concerning the measurement of bioenergetics of fish, Cooke et al. [23] reviewed the different ways of measuring this property in wild fish with sensor-equipped devices. These devices measured features such as heart rate, acceleration and image capture.

Another important property to measure in fish is their quality as food. For instance, smart sensors have been developed for measuring and estimating the quality of certain kinds of fish conserved in certain ice-refrigeration conditions [24].

Some works focus on monitoring fish for extracting either their location or their shape. More concretely, Saberioon and Cisar [9] presented a location tracking mechanism for multiple fish, based on light sensors. They used the Microsoft Kinect from outside the water, and used it for analyzing a limited water area (i.e., 60 × 30 × 10 cm^3^). In addition, Kawahara et al. [5] introduced a mechanism for capturing 3D shape models of fish from an underwater active stereo system. Their approach also integrated the use of an underwater camera. They obtained better results than an alternative mechanism that only used cameras.

There are some works that are more general about the measurement of different aspects of fish. In particular, Akyildiz et al. [2] reviewed the different mechanisms for collecting data in underwater environments. They reviewed works considering two-dimensional (2D) and three-dimensional (3D) distributions of underwater sensors. They mentioned some challenges in this area, such as the effective coordination of underwater sensors for providing meaningful information. In addition, Schaner et al. [6] presented a system for performing visual surveys of bottom fish. Their approach combined a camera with a depth sounder transducer for measuring the distance to the fish.

DIDSON sonars have been used for counting and measuring fish. In particular, Petreman et al. [14] applied DIDSON for counting fish in the inland waters. Their approach obtained similar results about the abundance of fish as nine people counting, but the total count of fish differed in 26%. They showed that the post-processing of fish counts from DIDSON raw data was labor-intensive, costly and inaccurate. In addition, Tušer et al. [25] proposed a mechanism for measuring the fish length by analyzing DIDSON data. They analyzed the reliability of their method considering different fish angles relative to the detector. The better estimations were obtained when the fish were perpendicular to the detector. Furthermore, Rakowitz et al. [26] proposed an approach based on DIDSON for analyzing the behavior of fish about avoiding a trawl net. In their study, they counted the fish for measuring the ratios of fish that properly avoided the trawl net considering some aspects such as fish length, distance to the trawl and avoidance fish speed. Therefore, DIDSON may be an appropriate sensor detection mechanism for counting fish according to the literature.

On the whole, most of the related works focus on the measurement of fish from sensors located in a very specific area. Nevertheless, most of the existing work do not consider the coordination of underwater sensors for measuring a wide area. In this line of research, Section 3 introduces an ABS that focuses on the definition and assessment of different strategies for measuring the amount fish in a wide area controlled by a grid of underwater sensors.

### 2.2. Multi-Agent Systems Related with Underwater Life

MASs have supported several analyses related with underwater life. In most cases, these systems were ABSs aimed at simulating different underwater scenarios.

To begin with, several ABSs modeled the influence of human actions on underwater life. In this way, people can estimate the repercussions of their actions with these ABSs. This could be helpful for stopping the activities that are estimated to have the worst impact on the corresponding ecosystems. For instance, Cenek and Franklin [27] presented an ABS model about the management of salmon fisheries using the coupled socio-ecological system models. They illustrated their approach with certain simulations in rivers, and showed how fishery influenced the behaviors of salmons. Their model estimated the possible outcomes of different fishery strategies. In addition, Gao and Hailu [28] introduced an ABS model that analyzed the impact of recreational fishing on the marine coral reef environment. They simulated different ways of choosing the site for fishing. They modeled the relationships between fish populations and fishing activities.

Some ABSs explicitly modeled the behavior of fish. These can explore their relations between the different species of fish and among the same ones. For example, Pais and Cabral [10] presented an ABS model for simulating the behavior of fish from different species. In this way, their system simulated the underwater fish census, as the amounts of the fish from the different species. They illustrated their approach with four fish species. They mainly simulated problematic behaviors such us excessive schooling, cryptic habits, shyness and boldness. Pereira et al. [29] introduced an approach for modeling ABSs inspired by ecological models. They illustrated their approach with the simulation of a fish farm. The fish were represented with agents that have goals, and these were sometimes against each other.

Several works introduce possible mechanisms for simulating underwater life. In particular, Helbing and Balietti [30] presented certain strategies to provide simulation capacities. Underwater life can be simulated with their approach following their guidelines for modeling individuals. Among other aspects, these guidelines recommend implementing birth, deaths, reproductions, competition, fighting ability, curiosity, mobility, carrying capacity and communication. In addition, Boids [31] is a simulation program that allows one to simulate the movement of fish schools in animations. Each fish is programmed with an autonomous behavior that consider other fish. The emergent animation behavior shows fish schools moving in a similar way to the reality.

Another group of works presented ABSs for analyzing the possible repercussions of environmental changes on the underwater life. In these works, the climate changes are one of the most relevant influential factors. In particular, Beltran et al. [32] introduced an ABS model for estimating the impacts of environmental changes on a top marine predator. They based their predictions on the energy balance of adult female individuals of this top predator species. Their approach simulated their anabolism. They associated their energy with their reproduction. In addition, Berman et al. [33] presented an ABS model for simulating the repercussions of people behaviors on the climate of Arctic ecosystems. Among others, these ecosystems support the life of fish.

Some MASs are related with the monitoring of underwater life and threats. In these cases, generally the agents are coordinated to perform the monitoring tasks. More concretely, Kadir and Arshad [34] developed a cooperative MAS for observing oceans based on a consensus algorithm. The goal of this MAS was to observe the coral reef environment. The agents were aimed at reaching consensus each time these had to decide where to head for continuing exploring the sea. The work of Jurdak et al. [21] had the objective of monitoring biosecurity threats in different environments. Among other applications, this approach was able to alert of aquatic pests. Their approach used aquatic robots with underwater sensors.

Nonetheless, to the best of the authors’ knowledge, there is not any ABS that provides a mechanism for defining and simulating different strategies about the measurement of the amount of fish. In this line of research, the next section introduces a novel ABS with this particular purpose for covering this gap of the literature.

## 3. ABS-FishCount

The goal of ABS-FishCount is to provide a simulation tool for defining and assessing different strategies for measuring fish with underwater acoustic sensors. ABS-FishCount relies on the pre-condition that all the underwater sensors are synchronized for performing the global counting of fish.

ABS-FishCount simulates the use of DIDSON sonar detectors. These detectors provide an image that is processed with CV techniques. Since CV techniques alone are normally not enough to accurately count the fish, ABS-FishCount assumes that these individual detectors would use some additional heuristic to correct the miscounts due to the overlapping. For example, similar heuristics have been previously defined for tuning the measurement of fish length from DIDSON data [14].

In particular, the current approach could be implemented using the DIDSON SV UL300 sonar model, which is distributed by the Sound Metrics corporation (Bellevue, WA, US). This model weights 7.9 kg in air and 1.0 kg in sea water. Its dimensions are 31.0 cm × 20.6 cm × 17.1 cm. These sonars can be connected to PCs with Windows 7 and Ethernet. Each DIDSON sonar and the corresponding PC could be included in a relative small floating/suspended station. According to their manufacturers, this DIDSON sonar has an effective range of 300 m for detecting objects.

Since the aforementioned DIDSON sonar only has a field of view of 29°, the implementation of the current approach could use the Sound Metrics X2 Rotator for rotating the DIDSON sonar for capturing information from 360°.

In the real world, some dynamic factors could affect the ability of the detectors of properly counting the fish. For example, it is a well-known fact that mobile sediment transport can hinder the detection capabilities of DIDSON sonars in some regions [14]. This can occur in rivers with high flows.

It is worth mentioning that counting fish can be really difficult in fish schools with a high density [35]. However, the current approach can be applied in fish species or situations whether the fish swim separately from each other or where fish schools have a very low density. For example, sharks and whales normally do not swim together in fish schools. In addition, the current approach will be able to be extended to incorporate the estimation count of fish schools in a wide sensor network, when the open challenge of this estimation mentioned by authors like De Kerckhove et al. [36] is achieved for a single fish school in the future.

The programming source code of the simulator is freely available from a public research dataset [37] not only to ensure the reproducibility of the experiments but also to allow other researchers to use it and extend it in different ways.

ABS-FishCount has been designed considering organizational modeling as commonly done in agent-based developments, as recommended in well-known agent-oriented methodologies such as ASPECS (an Agent-oriented Software Process for Engineering Complex Systems) [38]. In particular, this ABS was organized in two subsystems for, respectively, (a) underwater life simulation, and (b) underwater sensor network simulation. The interaction between these two subsystems is restricted only to the function that allows each sensor to obtain the relative local positions of fish in its detection range.

The agent-based design of the subsystem about underwater life simulation includes the following agent types:

- Fish agent: It represents a fish swimming in the water.
- Ecosystem agent: It simulates the creation and removal of fish in the ecosystem by spawning or destroying fish agents.

The subsystem about underwater sensor network simulation is designed with the following agents:

- Sensor agent: It represents an underwater sensor that can detect both fish and other underwater sensors in the nearby. It measures the amount of fish in the nearby. This measurement is intended to be overridden for obtaining different measurement strategies.
- Fish counter agent: This agent communicates with the underwater sensors through an underwater network. Its goal is to gather the information from all the sensor agents to provide an estimated measurement of the amount of fish in the monitored area.
- Water observer agent: This agent observes and saves the evolution of the simulation for later reporting it.
- Smart sensor agent: This agent extends the sensor agent to implement a smart way of measuring fish avoiding bias due to multiple detections of the same fish.
- Simple sensor agent: It extends the sensor agent to implement a basic way of measuring fish amount, and is used as control mechanism for the experimentation of the current work.

The presentation of ABS-FishCount is organized in several sections. In particular, Section 3.1 introduces the internal functioning of the subsystem about underwater life simulation. Section 3.2 formally presents the subsystem of underwater sensor networks. Section 3.3 introduces the way of defining measurement strategies and illustrates it with the definition of two strategies. Section 3.4 briefly introduces the implementation of the ABS, and presents its user interface (UI).

### 3.1. Subsystem of Underwater Life Simulation

The ecosystem was defined with the possibility of increasing and decreasing the number of fish. In particular, we defined a generic stochastic behavior following the recommendations of TABSAOND (a technique for developing ABS apps and online tools with nondeterministic decisions) [39].

In each simulation iteration (representing a day), the ecosystem can increase the number of fish or decrease it. This system simulates a daily change of the number of fish, as it is aimed at simulating crowded environments like fish farms. In uncrowded environments, the population evolution updates should be done in a lower frequency such as monthly. Generally, the increase simulates that fish are born. In open-water scenarios, the increase can also simulate that fish come from outside the monitored area. The decrease normally simulates that some fish can die or be eaten by other animals. It can also simulate that the fish are just taken by humans in fish farms, or that these are fished in open water. The decision about changing the number of fish is taken with the formula below following the recommendations of TABSAOND in each simulation iteration:

(1) $$d_{f}=\left\{\begin{array}{cc} \mathrm{increase}, & \mathrm{if}\;r\leq p_{i},\\ \mathrm{decrease}, & \mathrm{if}\;p_{i}<r\leq p_{i}+p_{d},\\ \mathrm{neutral}, & \mathrm{otherwise}, \end{array}\right.$$

where $d_{f}$ is the decision about the alteration of fish amount, *r* is a random number in the [0, 1] interval, $p_{i}$ is the probability of increasing, and $p_{d}$ is the probability of decreasing.

The user can establish different values of $p_{i}$ and $p_{d}$ from the UI, in order to test different ecosystems. In order to be consistent, the following condition must be fulfilled:

(2) $$p_{i}+p_{d}\leq 1,$$

where $p_{i}$ is the probability of increasing the number of fish in the ecosystem, and $p_{d}$ is the probability of decreasing it.

The probability $p_{n}$ of maintaining a neutral trend and not changing the number is implicitly defined from $p_{i}$ and $p_{d}$ with the following equation:

(3) $$p_{n}=1-\left(p_{i}+p_{d}\right),$$

where $p_{n}$ is the probability of not changing the number of fish in an ecosystem, and $p_{i}$ and $p_{d}$ are the probabilities of respectively increasing and decreasing this number of fish.

In case of increasing the number of fish, the new number of fish is calculated with the following equation:

(4) $$n_{f}=n_{f}^{\prime }+r_{i},$$

where $n_{f}$ is the number of fish of the current iteration, $n_{f}^{\prime }$ is the number of fish of the previous iteration, and $r_{i}$ is a random integer number in the $\left[0,N_{i}\right]$ interval. $N_{i}$ is the maximum number of fish increased per iteration.

If the ecosystem decides to decrease the number of fish, the new number of fish is calculated with the following equation:

(5) $$n_{f}=max\left(n_{f}^{\prime }-r_{d},0\right),$$

where $n_{f}$ and $n_{f}^{\prime }$ are the numbers of fish, respectively, in the current simulation iteration and the previous one, and $r_{d}$ is a random integer number selected from the $\left[0,N_{d}\right]$ interval. $N_{d}$ is the maximum number of fish that are decreased per simulation iteration. In case the result was a negative number, then the result is set to zero. In the equation, this is represented by calling the max function with the result and the zero number as parameters.

Since each simulation iteration represents a day, each fish can move to any other place from one day to another. Thus, in each iteration, the position of each fish is simulated randomly with the following equation:

(6) $$\vec{P_{f,k}}=\vec{\left(L_{x}+r_{x}\cdot w,L_{y}+r_{y}\cdot h\right)},$$

where $\vec{P_{f,k}}$ is the position of each *k* fish, $L_{x}$ and $L_{y}$ are the minimum limits of the monitored water, respectively, in the *x* and *y* axes, *w* and *h* are, respectively, the width and height of the monitored water area, and $r_{x}$ and $r_{y}$ are two random numbers in the [0,1] interval.

### 3.2. Subsystem of Underwater Sensor Networks

This approach simulates stations with underwater sonar sensors that can detect fish [40]. These stations also have sonar sensors that can also detect other stations with sonar sensors. These stations are suspended in the water but tied with ropes.

Regarding the detection of fish, the sensors can detect some of the fish within a range $R_{f}$, considering an instrumentation error with a probability of $p_{e}$. The sonar detection of fish is simulated with the following equation:

(7) $$s_{f}\left(i,j\right)=\left\{\begin{array}{cc} \mathrm{detect}, & \mathrm{if}\;|((\vec{P_{s,i}}-\vec{P_{f,j}}|\leq R_{f})\wedge \left(r>p_{e}\right)),\\ \mathrm{not}\;\mathrm{detect}, & \mathrm{otherwise}, \end{array}\right.$$

where $s_{f}\left(i,j\right)$ is whether the sensor *i* detects the fish *j*, $\vec{P_{s},i}$ is the position of the underwater sensor *i*, $\vec{P_{f,j}}$ is the position of the fish *j*, $R_{f}$ is the maximum range distance in which a fish can be detected with a sonar, $p_{e}$ is the probability that an instrumentation error occurs, and *r* is a random number in the [0, 1] interval.

The current approach uses a heuristic that mitigates the repercussion of the instrumentation error. In particular, this heuristic considers the instrumentation errors when estimating the amount of fish in a sensor range with the following equation:

(8) $$n_{f}\left(i\right)=\frac{\sum_{j\in F}s_{f}\left(i,j\right)}{1-p_{e}},$$

where $n_{f}\left(i\right)$ is the amount of fish estimated by the *i* sensor, *F* is the set of the simulated fish, and $s_{f}\left(i,j\right)$ and $p_{e}$ have the meanings previously introduced. Notice that $s_{f}\left(i,j\right)$ is counted as one for the detections and as zero in the other cases.

In the underwater sensor estimation strategies where the fish are treated individually considering their positions, each fish is counted with its proportional part considering the instrumentation error with the following equation:

(9) $$s_{f}^{\prime }\left(i,j\right)=\frac{s_{f}\left(i,j\right)}{1-p_{e}},$$

where $s_{f}^{\prime }\left(i,j\right)$ is the individual estimation measure of the *j* fish by the *i* sensor, $s_{f}\left(i,j\right)$ is evaluated as one if the the *j* fish is detected by the *i* sensor and as zero otherwise, and $p_{e}$ is the probability of the instrumentation error.

The underwater sonar sensors can detect other sensors in other stations in a different range $R_{s}$. The detection of another sensor is expressed with the following equation:

(10) $$s_{s}\left(i,j\right)=\left\{\begin{array}{cc} \mathrm{detect}, & \mathrm{if}\;|\vec{P_{s,i}}-\vec{P_{s,j}}|\leq R_{s},\\ \mathrm{not}\;\mathrm{detect}, & \mathrm{otherwise}, \end{array}\right.$$

where $s_{s}\left(i,j\right)$ determines whether the sensor *i* senses the sensor *j*, $P_{s,i}$ and $P_{s,j}$ are, respectively, the positions of sensors *i* and *j*, and $R_{s}$ is the maximum range distance in which two sensors can detect each other.

There are several ways of measuring fish considering the fish and/or other sensors, as it will be introduced later in Section 3.3. However, in the real world, these sensors have a low error rate in which these can miss detecting a fish or wrongly detect another object as a fish. In order to model these errors, the system converts the theoretical measure into a more realistic measure with the following equations based on TABSAOND:

(11) $$d_{e}=\left\{\begin{array}{cc} \mathrm{yes}, & \mathrm{if}\;r\leq p_{e},\\ \mathrm{no}, & \mathrm{otherwise}, \end{array}\right.$$

where $d_{e}$ simulates the decision about whether simulating an error, *r* is a random number in the [0,1] interval, and $p_{e}$ is the probability of introducing an error.

In case of introducing an error, the following equation is used for simulating this error:

(12) $$m_{r}=\left\{\begin{array}{cc} m_{t}+1, & \mathrm{if}\;r\leq p_{FP},\\ max(m_{t}-1,0), & \mathrm{otherwise}, \end{array}\right.$$

where $m_{r}$ is the realist measure that considers sensor errors, $m_{t}$ is the theoretical measure in which the sensors are assumed to not have sensing errors, *r* is a random number in the [0,1] interval, and $p_{FP}$ is the probability that an error is a false positive (i.e., a wrong detection of a fish), provided that an error has been produced. Otherwise, the error would be a false negative (a fish that is not detected). The formula guarantees that the realistic measure is never a negative number by means of the max function. Provided that an error occurs, probability $p_{FN}$ about occurring a false negative is implicitly determined with the following equation:

(13) $$p_{FN}=1-p_{FP}.$$

The underwater sensors are tied with ropes in the monitored water area. Some wires are attached to these ropes for supporting the communication infrastructure of the sensor network. This wired communication facilitates the synchronization among sensors and the collection of the sensor results. The monitored area can be either a fish farm or an open-water area. The ropes grid maintains each sensor around a position referred as its original position. The original positions of the sensors are established for each sensor with the following equation:

(14) $$\vec{Po_{s,i,j}}=\vec{\left(L_{x}+I_{x}/2+i*I_{x},L_{y}+I_{y}/2+j*I_{y}\right)},$$

where $\vec{Po_{s,i,j}}$ is the original position of the sensor situated in the (i,j) relative order position of the grid, $L_{x}$ and $L_{y}$ are the minimum limits of the monitored water are respectively in the *x* and *y*-axes, and $I_{x}$ and $I_{y}$ are the interval distances between the sensors respectively to each axis. After placing the sensors, $L_{x}$ and $L_{y}$ normally represent some approximate coordinate bounds of the sonars’ effective range. The following equations calculate these intervals from (a) the numbers of rows and columns entered by the user, and (b) the width and height of the monitored water area:

(15) $$I_{x}=w/n_{c},$$

(16) $$I_{y}=h/n_{r},$$

where *w* and *h* are, respectively, the width and height of the monitored water area, and $n_{c}$ and $n_{r}$ are, respectively, the numbers of rows and columns of the grid of sensors.

However, the water currents can move the sensors from its original position to another position up to certain distance limit $L_{s}$. Since each simulation iteration represents a day, the position from one day to another can be completely different within the range. In each day, the current position of each sensor is calculated with the following equation:

(17) $$\vec{P_{s,i,j}}=\vec{Po_{s,i,j}}+r\cdot L_{s}\cdot \vec{\cos\left(r_{a}\right),\sin\left(r_{a}\right)},$$

where $\vec{P_{s,i,j}}$ is the current position of the sensor $s_{i,j}$, $\vec{Po_{s,i,j}}$ represents its original position, *r* is a random number in the [0,1] interval, $L_{s}$ is the maximum distance limit in which the sensor can move, and $r_{a}$ is a random angle in the [0,2×PI] interval.

### 3.3. Strategies of Underwater Sensors for Counting Fish

The strategies of counting fish can be defined by extending sensor agent and overriding the “Measure” method. The sensor agent type has methods visible for its subtypes, and these methods allow obtaining the location information about the nearby fish and other sensor stations separately.

The simplest strategy is that each sensor counts the nearby fish and then sends this measure to the fish counter agent. This agent sums these measurement values. In particular, this simple strategy can be defined with the following equation:

(18) $$c_{c}=\sum_{k=0}^{N}c_{c,k},$$

where $c_{c}$ refers to the count of fish in which the simple strategy is used as control mechanism, *N* is the number of sensors, and $c_{c,k}$ is the number of fish that are estimated by the sensor *k*, previously referred to as $n_{f}\left(i\right)$.

In particular, the count of each sensor $c_{c,k}$ can be defined with the following formula:

(19) $$c_{c,k}=\left|F\right|,$$

where *F* is the set of the estimated fish following the detection mechanism explained in Section 3.2.

Since the sensors are nearly distributed homogeneously in water space, if the detection distance of fish $R_{f}$ causes the areas detected by sensors to not overlap too much and cover most of the area, the results can be approximately correct.

Moreover, we developed another strategy for counting fish, which will be referred to as smart strategy from now on. In this case, the global mechanism of the fish counter agent is similar to the previous case, as it sums the measures of the sensors. However, sensor agents count fish in a different way. The global count is defined with the equation below:

(20) $$c_{s}=\sum_{k=0}^{N}c_{s,k},$$

where $c_{s}$ is the total fish count with the smart strategy, *N* has the same meaning as before, and $c_{s,k}$ is the number of fish counted smartly by sensor *k*.

In the smart strategy, each sensor counts each fish as one divided by the number of sensors that can observe it including itself. For knowing which sensors observe each fish, it calculates the distance between the fish and the other sensors. This mechanism works thanks to the fact that sensor stations can know the location of other sensor stations that are at least two times further than the detected fish. In particular, this smart count $c_{s,k}$ of each sensor *k* is calculated with the formula below:

(21) $$c_{s,k}=\sum_{f_{j}\in F}1/|\{s_{i}\in S:|\vec{P_{s,i}}-\vec{P_{f,j}}|\leq R_{f}\}|,$$

where *F* is the set of the fish estimated by the sensor, *S* is the set of the sensors detected by the sensor *k* including itself, $\vec{P_{s,i}}$ is the position of each sensor $s_{i}$ of the *S* set, $\vec{P_{f,j}}$ is the position of each fish $f_{j}$, and $R_{f}$ is the maximum range distance in which sensors are assumed to detect fish.

It is worth noting that in the real world there is no way of accurately validating the effective range of fish detectors. In addition, in very dynamic environments, the ranges cannot be assumed to be perfect and constant circles/spheres as maximum range distance may vary even for the same fish detector. In order to address these issues, the range distance threshold $R_{f}$ is recommended to be set to a distance in which almost surely the detector can detect fish (i.e., below the common actual maximum range distance). Hence, the detector will probably detect fish further than $R_{f}$. In those cases, the detector will check the distance. Since the corresponding fish will be outside of its assigned area, the detector will not count this fish. In this way, the proposed smart strategy can be properly applied in the real world, since the detectors actually detect the fish up to the $R_{f}$ distance and only consider fish up to this distance.

In this manner, the fish that are only detected by one sensor are counted as one. However, if a fish is detected by several sensors, as it is an overlapped monitored area, then the fish is counted in portions, where each sensor counts its corresponding portion.

### 3.4. Implementation and User Interface

ABS-FishCount has been developed following the Process for developing Efficient ABSs (PEABS) [41] for conceiving the general design of its agents and implementing these into a simulator. In particular, we have used a Unity 3D engine with C# as the programming language. This engine was selected to facilitate the creation of a visual and intuitive interface. C# was selected as it fulfills both the possibility of integrating with the selected engine and the fact of being object-oriented. The latter is useful for (a) implementing PEABS in a straightforward way and (b) for having the possibility of defining subtypes of agents by inheriting the classes of the corresponding agents.

In the current work, the definition of subtypes agents is useful for defining different types of underwater sensor agents implementing different measurement strategies. More specifically, these strategies can be implemented by extending the “Sensor Agent” class and overriding the “Measure” method. The smart sensor agent can be used as an example for the definition of sensor strategies. The parent sensor agent class provides the necessary methods for observing both the nearby fish and sensors with their positions in the local coordinates.

**Figure 1:**
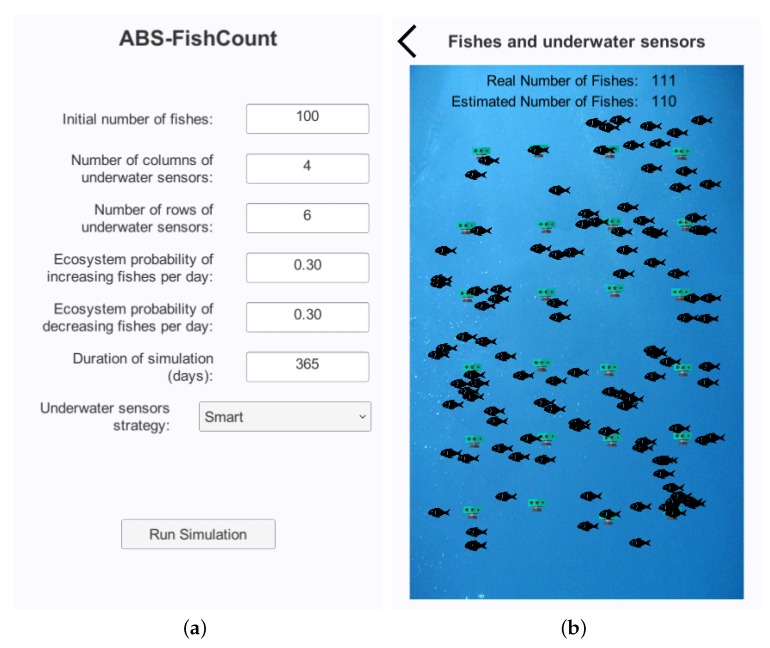
User interface (UI) of ABS-FishCount (an agent-based simulator of underwater sensors for measuring the amount of fishes). (**a**) input screen; (**b**) output screen.

Figure 1 shows the UI of the ABS-FishCount application. More concretely, Figure 1a presents the screen where the user enters the input parameters. These include the initial number of fish of the simulation and the number of rows and columns of the grid of underwater sensors. Users can also configure the stochastic behavior of the ecosystem regarding the alteration of the number of fish. This behavior is defined with two probabilities respectively concerning to (a) the appearance of new fish, either by being born or coming from outside the monitored area, and (b) the decrease of fish either by dying, being eaten or leaving the monitored area. The duration of the simulation is specified with any number of days. Finally, different underwater sensors’ strategies can be selected from a dropdown list.

Figure 1b shows the screen that contains the final results of the simulation. It indicates the real number of fish in the ecosystem from the count of actual fish agents. It also indicates the estimated number of fish obtained from the collective measurement from the sensor agents following the particular strategy. This screen presents a graphical representation of the simulated water area. Both the sensor agents and the fish agents are represented with icon images in the corresponding positions. Since fish swim randomly, these are distributed in the space without any specific pattern. However, sensor agents have the restriction that they are originally attached to a position although this attachment tolerates their movement up to a certain threshold distance. In this execution example, one can observe that sensor agents conform to a grid, but the sensors are not in the exact positions of the grid intersections because of these slight position variations.

The presented tool also outputs text files with the evolution of the simulations, indicating the number of fish and the estimation from the measurement of sensors. In this manner, we were able to further analyze the results of the simulation evolutions. Section 4.2 shows examples of these simulation evolutions.

## 4. Experimentation

### 4.1. Experimentation Setup

In order to experience the presented simulator and approach, we have compared the two proposed strategies in three different ecosystems types, so that the results can be generalized to different ecosystems. Table 1 shows the configuration of the input parameters for representing each ecosystem type. These ecosystem types are referred to as “neutral”, “increasing” and “decreasing”, and their names refer to the trends of the number of fish. The neutral ecosystem does not vary the number of fish significantly in the long term, although it has a lot of small variations during the simulation. The increasing ecosystems are the ones in which normally fish are born more frequently than the dying or being eaten. The decreasing ecosystems represent the opposite, i.e., when fish die or are eaten more often than they are born. In this experimentation, increasing ecosystems start with a low number of fish (i.e., 15), while we started ecosystems with decreasing trends with a relative high number of fish (i.e., 250).

All of the simulations were performed in a 4 × 6 grid of underwater sensors. These sensors were uniformly distributed in a fish farm controlled area of 450 × 700 m^2^. Each sensor was able to sense fish in a radium of 75 m and other sensors in any distance up to 150 m. Each sensor had the restriction of not being further away than 15 m from its original position of the corresponding grid intersection. We selected the value of 75 m as the range distance of DIDSON sonars, since this distance was reported in the work of Song [42]. We chose a position error of 15 m as other works in the underwater field such as the one by Vaganay et al. [43].

The sensor agents had a probability of failing of 0.001. In case of failing, the corresponding error had a probability of 0.40 of being a false positive, i.e., the sensor would wrongly detect a fish that does not exist. In the other error cases, the system missed detecting a fish. The ecosystem was configured to have alterations of up to three fish per day.

### 4.2. Comparison of Simulation Evolutions

**Figure 2:**
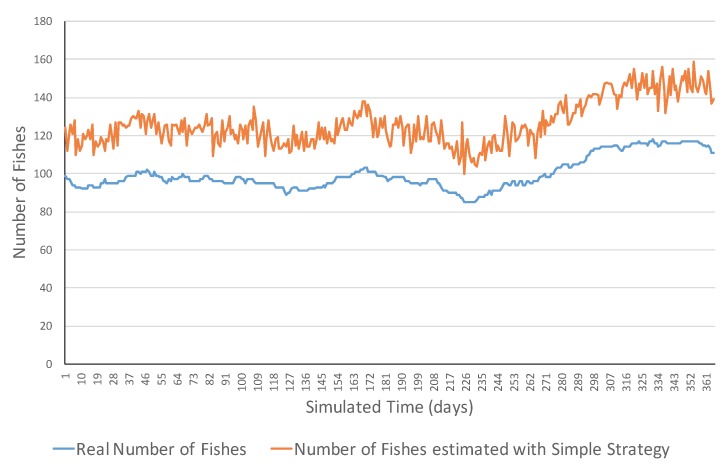
Simulation evolution of a neutral ecosystem with the simple strategy.

This section presents examples of the simulation evolutions of the different combinations of ecosystem types and strategies. Figure 2 shows an example of simulation evolution of the fish count in a neutral ecosystem and its estimation with the simple strategy of underwater sensors. As one can observe, there are not notorious changes in the fish count in the long term although there are different variations within the evolution which is realistic and normally similar to real ecosystems. The fish count values ranged in the interval of [85, 118]. In this simulation, one can observe that the estimated count of fish with the simple strategy was normally higher than the real number of fish, with errors in the range of [12, 42] in the number of fish. This bias of the counting was probably due to the fact that some fish were sensed by more than one sensor, and consequently are counted more than once.

**Figure 3:**
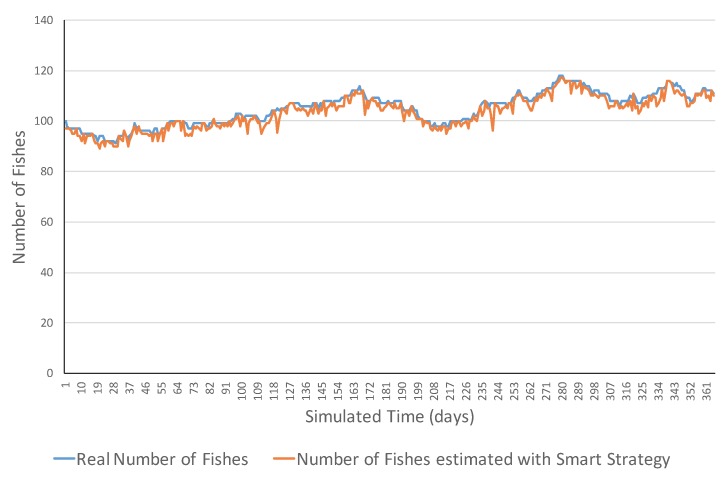
Simulation evolution of a neutral ecosystem with the smart strategy.

Figure 3 shows an example of an evolution in the same ecosystem type, but this time the measurement of fish was performed with the smart strategy. In this case, the estimated measurement values were much closer to the real ones probably. This was reflected in the fact that the absolute errors ranged in the [0, 11] interval. Moreover, in this case, the mean squared error (MSE) was 5.20, while, in the previous case with the simple strategy, was 742.67. The errors of the smart strategy were lower probably because underwater sensors properly addressed the cases in which a fish was sensed by several sensors by the proportional count of each fish. The small errors can be due to the simulated errors of underwater sonar sensors.

**Figure 4:**
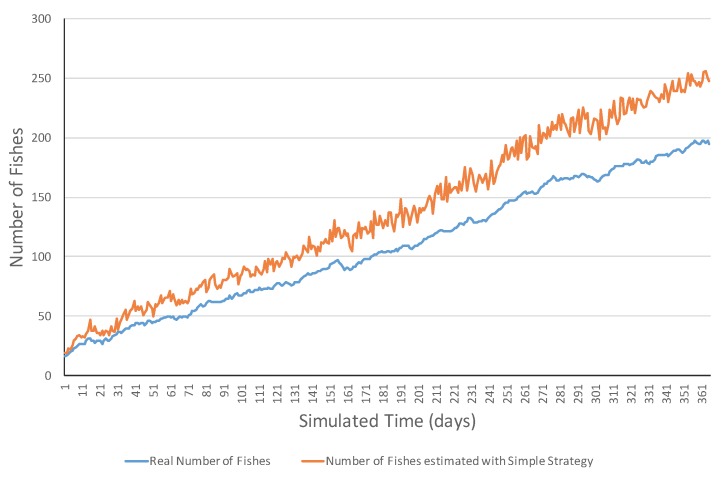
Simulation evolution in an increasing ecosystem with the simple strategy.

**Figure 5:**
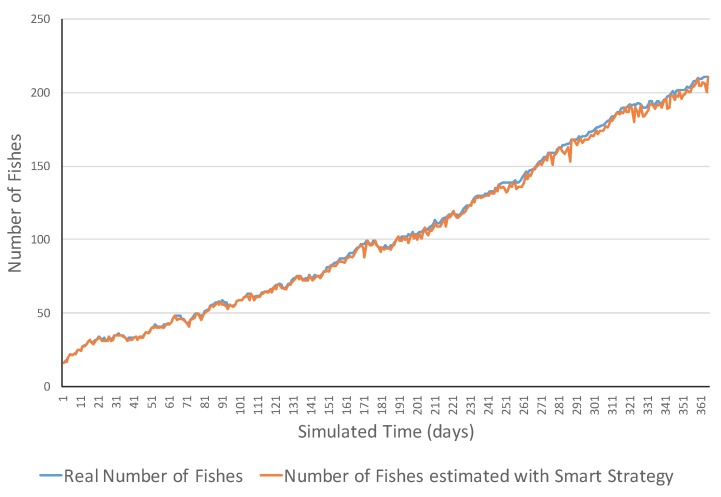
Simulation evolution of an increasing ecosystem with the smart strategy.

Figure 4 presents an example of an increasing ecosystem. This ecosystem showed realistic variations in the short term including both increasing and decreasing ones. However, in the long term, the number of fish increased from 15 fish to 195 fish. The MSE error was 1075.31 and errors ranged in the [2, 62] interval. Figure 5 shows a simulation example of the same ecosystem but using a smart strategy. One can observe that the estimations were much closer to the real values. This was also reflected in the much lower MSE (i.e., 6.77 fish), and all the errors belonged to the [0, 13] interval.

**Figure 6:**
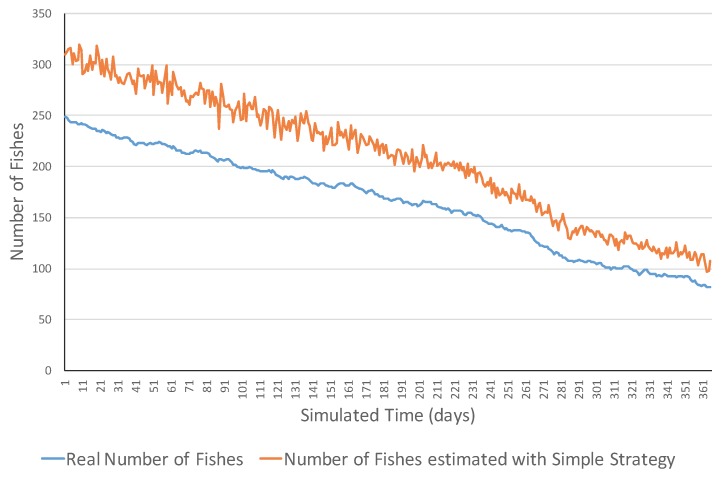
Simulation evolution of a decreasing ecosystem with the simple strategy.

**Figure 7:**
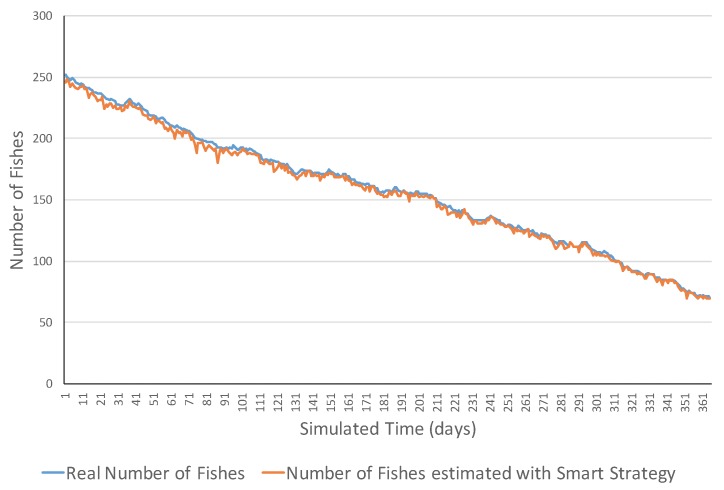
Simulation evolution of a decreasing ecosystem with the smart strategy.

Figure 6 shows the evolution of a decreasing ecosystem with the simple strategy in the measurement of fish, while Figure 7 presents a simulation example of the same ecosystem type with the smart strategy. The results of this ecosystem were very similar to the previous ones. The smart strategy obtained lower errors than the simple strategy (MSE of 2188.99 compared to 10.60). The difference of this ecosystem type was that the real number of fish decreased from 250 to, respectively, 82 and 72 fish. Notice that this approach uses the TABSAOND technique with the nondeterministic decisions, and the final results may slightly vary from one execution to another with the same input parameters.

The current approach was based on the assumption that underwater sensors can be synchronized as previously mentioned in Section 3. In addition, the Unity 3D engine follows a synchronous approach based on a step-by-step time progress synchronized to the 3D scene rendering. Thus, the presented results of this section and the next one are only meaningful for the real-world scenarios in which this synchronization can be achieved among the underwater sensors.

### 4.3. Comparison of Final Results

**Figure 8:**
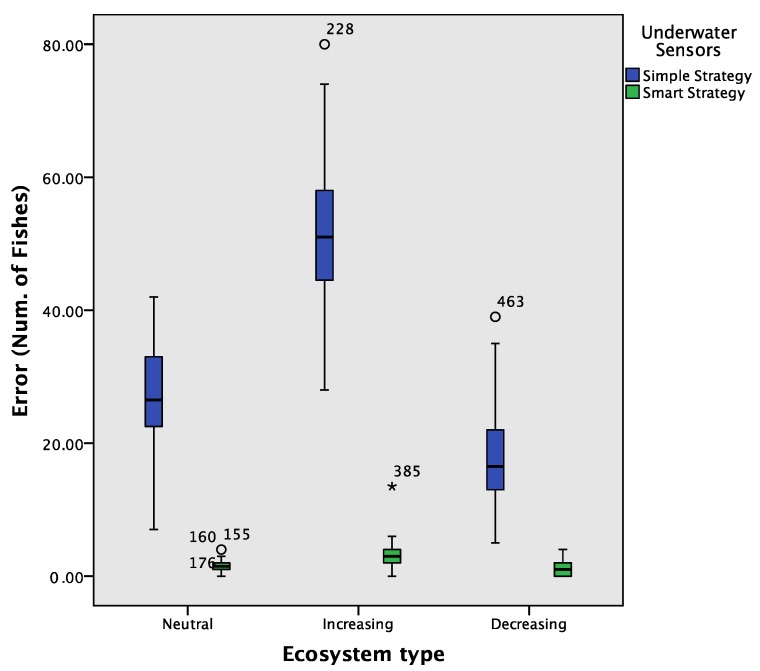
Comparison of errors with a boxplot considering the results of 100 simulations for each ecosystem and each underwater sensors strategy.

The current work also compares the final results between the two underwater sensor measurement strategies with the three ecosystem types. In order to avoid bias due to the nondeterministic behavior of the ABS, the simulator was executed 100 times for each ecosystem type and each underwater sensors strategy. The errors were measured as the absolute differences between the real number of fish and the estimated ones. Figure 8 shows a boxplot chart that compares these mean errors at the end of the simulations between the different combinations of ecosystem types and strategies. One can observe that the smart strategy obtained average errors much lower than the simple one.

In order to determine whether the differences of errors between both strategies were statistically significant, we applied the Welch’s *t*-test, also known as the unequal variance *t*-test. We selected this test as it is robust for comparing means even if the equality of variances cannot be assumed. We also applied the Brown–Forsythe test as it is also robust for comparing means in these cases. Table 2 shows the results of these statistical tests. Both tests coincided in determining that the differences of errors between these two strategies were statistically significant, with a significance level of 0.001. Thus, the smart strategy is statistically significantly more accurate in measuring the amount of fish than the simple strategy.

In order to measure the improvement of the smart strategy over the simple strategy, we calculated the Cohen’s d effect size following the guidelines of Cohen [44]. We obtained that this effect size was 2.55 between the errors of the two strategies. This effect size is very large according to the interpretation of Rosenthal [45].

## 5. Conclusions

The current work has presented the novel ABS-FishCount simulator that allows one to define and assess different strategies for measuring fish from a set of underwater sonar sensors. It focuses on the implicit coordination for obtaining an accurate measurement from the individual measures of the underwater sensors. Their possible applications include (a) the measurement in fish farms to anticipate the fish production for accurately knowing the fish amount that can be sold in advance, and (b) the tracking of fish amounts in open-water to estimate the evolution of real ecosystems. The current approach has been illustrated by simulating two different strategies of fish measurement by means of a grid of underwater sensors. One was simple and was used as the control experiment. The other strategy was referred to as the smart one, and used an implicitly coordinated measurement based in the observation of other sensors. The presented simulator is open-source to (1) allow other researchers to define and assess different fish measurement strategies and (2) to extend the simulator, for example by including new fish agent behaviors or adding other ecosystem evolution kinds.

In the near future, we plan to develop and assess more measurement strategies with ABS-FishCount. In addition, the simulator can be extended for considering fish sizes to measure the total weight of fish. This can be useful for fish farms. In this case, the tool will simulate the growth of fish. For now, ABS-FishCount simplifies the model into a 2D world. However, this could be easily generalized for a 3D environment. Our future work includes the development of another simulator of underwater sensors for tracking specific fish in real-time. In this case, the timing will be different as each simulation iteration may represent a second instead of a day. In this case, the evolution of the simulation will be presented in an animation-like visualization in which the user will be able to see the fish moving. In the long term, the strategies implemented in ABS-FishCount and its extensions are planned to be integrated in a real underwater sensors network to assess their actual utility in the real world.

## Figures and Tables

**Table 1:** Input parameters of the simulator for the different ecosystem types.

	Neutral	Increasing	Decreasing
Initial number of fish	100	15	250
Number of columns of underwater sensors	4	4	4
Number of rows of underwater sensors	6	6	6
Ecosystem probability of increasing fish per day	0.30	0.75	0.25
Ecosystem probability of decreasing fish per day	0.30	0.25	0.75
Duration of simulation (days)	365	365	365

**Table 2:** Robust tests of equality of means for comparing the errors between the two strategies.

	Statistic ${}^{a}$	df1	df2	Sig.
Welch	977.017	1	305.170	0.000 **
Brown–Forsythe	977.017	1	305.170	0.000 **

${}^{a}$ Asymptotically F distributed; ** Statistically significant with a significance level of 0.001.
